# Cross-Calibration of iDXA and pQCT Scanners at Rural and Urban Research Sites in The Gambia, West Africa

**DOI:** 10.1007/s00223-023-01071-6

**Published:** 2023-03-02

**Authors:** Mícheál Ó Breasail, Ramatoulie Janha, Ayse Zengin, Camille Pearse, Landing Jarjou, Ann Prentice, Kate A. Ward

**Affiliations:** 1 Population Health Sciences, Bristol Medical School, University of Bristol, 1-5 Whiteladies Road, Bristol, BS8 1NU UK; 2 MRC Nutrition and Bone Health Research Group, Clifford Allbutt Building, University of Cambridge, Cambridge Biomedical Campus, Hills Road, Cambridge, CB2 OAH UK; 3 MRC Unit The Gambia at London, School of Hygiene and Tropical Medicine, Banjul, Gambia; 4 Department of Medicine, School of Clinical Sciences, Faculty of Medicine, Monash Medical Centre, Nursing and Health Sciences, Monash University, Clayton, VIC Australia; 5 MRC Lifecourse Epidemiology Centre, University of Southampton, Southampton General Hospital, Tremona Road, Southampton, SO16 6YD UK; 6 MRC Epidemiology Unit, University of Cambridge, Cambridge, UK

**Keywords:** Densitometry, DXA, pQCT, Cross-calibration, Body composition

## Abstract

Between-scanner differences in measures of bone and body composition can obscure or exaggerate physiological differences in multi-site studies or the magnitude of changes in longitudinal studies. We conducted a cross-calibration study at two bone imaging centres in The Gambia, West Africa where DXA (dual-energy X-ray absorptiometry) and pQCT (peripheral Quantitative-Computed Tomography) are routinely used. Repeat scans were obtained from 64 Gambian adults (58% Male) aged Mean(SD) 30.9 (13.5) years with Mean(SD) body mass index (BMI) 21.7 (4.0) kg/m^2^, using DXA (GE Lunar iDXA, whole body [WB], total hip [TH], lumbar spine [LS]) and pQCT (Stratec XCT2000L/XCT2000, tibia 4%, 50% sites). Between-scanner differences were tested using paired t tests (*p* < 0.05). Between-scanner correlation was explored with linear regression, and cross-calibration equations derived. Bland–Altman analysis investigated machine trend/bias. When differences were detected (*p* < 0.05), cross-calibration equations were applied to urban values, with t tests and Bland Altman analysis repeated. Between-scanner differences exceeded the predefined level of statistical significance (*p* < 0.05) for WB aBMD and BA; all pQCT measures vBMD, BMC, cortical cross-sectional area (CSA) and stress–strain index (SSI). Between-scanner correlation was high (*R*^2^:0.92–0.99), except pQCT Mu.Den (*R*^2^ = 0.51). Bland Altman plots indicated bias increased with increasing BMD. Cross-calibration equations attenuated all between-scanner differences and systematic bias. Cross-calibration, particularly of pQCT scanners, is an important consideration in multi-site studies particularly where between population comparisons are intended. Our experiences and findings may be generalisable to other resource-limited settings where the logistics of sourcing parts and in-country repair may result in lengthy scanner downtime.

**Supplementary Information:**

The online version contains supplementary material available at 10.1007/s00223-023-01071-6.

## Introduction

In musculoskeletal research, multi-site studies are of particular importance as certain primary outcomes require quite large sample sizes. Multi-centre studies are valuable as the data obtained allow for geographic representation and increased sample size, together allowing for more generalisable inferences than those of a single-site study. However, even with carefully calibrated musculoskeletal imaging modalities, between-scanner differences are acknowledged as a potentially serious problem when comparing both bone and body composition outcome measures [1–8]. While the magnitude of between-scanner differences varies, due to technological (e.g. calibration to read zero at the density of water or fat) [9] and environmental factors (e.g. temperature) [10], with comparability likely to be lower where the model of, or manufacturer of, the technology differs. However, even devices of the same model may differ at the extremes of their measurement range. International Society of Clinical Densitometry (ISCD) guidelines detail how to address and minimise potential differences between scanners when adding hardware or systems, through cross-calibration [11]. This approach is also applicable to multi-site studies and facilitates the comparison of measurements obtained across several research centres.

This paper presents our experience of cross-calibrating dual-energy X-ray absorptiometry (DXA) and peripheral quantitative-computed tomography (pQCT) scanners at two bone imaging facilities in The Gambia, West Africa. We undertook this work to ensure that between-scanner differences do not attenuate genuine physiological differences between our research populations in rural and urban settings. In addition, The Gambia is currently undergoing marked epidemiological transition with high rural to urban migration, cross-calibration allows us to retain participants in longitudinal studies even where they have previously participated in studies conducted at the rural field station and have since migrated to the urban coastal region. This reduces unnecessary loss to follow up and enables studies of longitudinal changes in muscle and bone measures to be conducted. Until now we have where necessary in longitudinal studies [12] made use of phantoms, such as the European Forearm Phantom (EFP) [13], to correct pQCT bone density-based outcomes but this has precluded the correction of body composition measures. Moreover, while it is accepted that bone and body composition vary between populations and efforts have been undertaken to ameliorate the impact of this on DXA [14], there are a lack of data from sub-Saharan African populations. Research has previously highlighted differences in bone [15, 16] and body composition [17] between Gambians and UK adults, as such it remains uncertain whether the application of correction equations from different populations are appropriate in this context.

Therefore, the aims of this in vivo study were (a) to determine, across a range of bone and body composition measures, whether between-scanner differences were present and whether between-scanner bias existed across a range of bone mineral densities and body compositions; (b) if so, calculate appropriate cross-calibration corrections for the respective pairs of DXA and pQCT scanners in the urban and rural sites in The Gambia.

## Methods

### Participants

Men and women aged 18–75 years living in The Gambia were eligible for recruitment. As the distance between both research sites is quite large, potential participants living in the vicinity of the MRC Unit The Gambia at London School of Hygiene and Tropical Medicine (LSHTM)—Fajara (urban setting) who had migrated from, or had family ties to, the area close to the MRC Unit The Gambia at LSHTM—Keneba in the rural Kiang West region were contacted to facilitate participation. Potential participants were identified by the Kiang West demographic surveillance system (KWDSS) [18]. Additionally, those who had previously taken part in research at the urban site that consented to being contacted about future research studies were invited to participate in the study. Eligibility criteria included being aged 18–75 years, able to provide informed consent, having migrated from Kiang West, and willingness to travel to MRC Keneba for repeat measurements. Exclusion criteria included women of reproductive age who were or were unsure whether they were pregnant/lactating, history of metabolic bone disease, unable to give informed consent, and those with metal implants within their body.

### Sample-Size

ISCD guidelines recommend a minimum of 15 participants be scanned at least twice for cross-calibration [11]. As these guidelines are based primarily on measurements obtained in Caucasian populations, we aimed to recruit a higher number of participants to account for a potential greater variance in Gambian bone density and body composition. We chose a minimum sample size of 60 participants to ensure that there were enough data points to derive robust correction factors and to account for attrition and exclusion of scans due to movement or other artefacts. Due to the logistics of transporting participants approximately 150 km between the two research facilities, we pragmatically aimed to ensure all study visits took place within a three-day window, but no later than 3 weeks. Participants in this study were representative of those normally scanned at these facilities.

### Anthropometry

Anthropometry was collected at the first visit. Height (m) was obtained without footwear to the nearest 0.01 m by wall-mounted stadiometer (Seca GmbH, Hamburg, Germany). Weight (kg) was measured to the nearest 0.1 kg using a digital scale, with participants wearing light clothing without footwear (Seca GmbH). Tibia length (mm) measurements were obtained to the nearest 1.0 mm using a tape measure from the distal edge of the medial malleolus to the tibial plateau.

### Densitometry

As per ISCD guidance, we designated one of each scanner pair as our index (‘gold standard’) device, in each case, this was the scanner at the rural site. This decision was based on the longer use of the rural imaging facility, the greater number of scans acquired there, and that many participants in ongoing longitudinal studies had their baseline scans obtained there. Calibration of all DXA and pQCT systems was performed on a routine basis using manufacturer’s phantoms: daily quality-assurance scans and weekly quality-control scans were performed throughout the study period to test scanner performance.

#### DXA

GE Lunar iDXA (Waltham, MA, USA) scanners were used at each facility, and each participant was scanned at the whole body (WB), total hip (TH), and lumbar spine (LS, L1–L4). All image analysis was conducted in the manufacturer’s software Encore version 15 (GE, Waltham, MA, USA). Bone measures of interest for all sites were areal BMD (aBMD, g/cm^2^), BMC (g), and bone area (BA, cm^2^). Body composition measures from WB scans were lean mass (LM, g) and fat mass (FM, g). Pairs of DXA scans not obtained using the same scan mode (i.e. standard vs thin) were not included in our analyses. The short-term precision at the rural site, measured as coefficient of variation (CV%) of duplicate measurements in 70 Gambian adults was < 1% for all sites for aBMD. The same team operates across both research sites, with all DXA scans scrutinised by a single-team member (RJ) prior to export for analysis.

##### pQCT

Scans were obtained using a Stratec XCT2000L (Stratec Medizintechnik, Pforzheim, Germany) at the rural site and a Stratec XCT2000 at the urban site. Differences between these scanners are minimal and relate to the scan range (XCT2000, 230 mm; XCT2000L 400 mm). Scans of the non-dominant tibia were performed with a voxel size of 0.5 mm and slice thickness of 2 mm at 4 and 50% of the limb length proximal to the distal endplate. CT scan speed was 30 mm/s, and scout view scan speed was 40 mm/s. Scans were processed using the manufacturer’s software (Stratec XCT version 6.2): at the 4% site CALCBD analysis contour mode 1, peel mode 1 at a threshold of 180 mg/cm^3^ was used with trabecular bone being defined as the inner area of 45% of the total cross-sectional area (Tot.A) to measure total volumetric bone mineral density (Tot.vBMD) and trabecular vBMD (Tb.vBMD). At the 50% site, a threshold of 710 mg/cm^3^ was selected in conjunction with CORTBD separation mode 1 and measures of cortical bone mineral content (BMC), cortical volumetric bone mineral density (Ct.vBMD), cortical cross-sectional area (Ct.A), and cortical thickness (Ct.Th) derived. Here, Tot.A was defined at a threshold of 280 mg/cm^3^. Muscle cross-sectional area (CSMA) was quantified using a threshold of 40 mg/cm^3^ and a muscle smoothing filter (F03F05). Stress–strain Index (SSI), an estimate of bone strength, was obtained at a threshold of 280 mg/cm^3^ using cortmode 1. Muscle density (Mu.Den) was assessed using a threshold of 100 mg/cm^3^ and filter F03F05. Scans were qualitatively graded by visual inspection by a single-team member (MÓB) to assess their suitability for longitudinal analysis: scan slices with excessive movement or other artefacts and scout views that did not match longitudinally were excluded. Studies at our site found the inter-operator pQCT precision to be 0.3 to 1.8% for bone outcome measures at the tibia by performing two repeat scans on 30 Gambian participants. All scans were acquired by the same imaging team which operates across the two research sites.

### Statistical Analysis

All analyses were performed in R (version 4.0.3); statistical significance was set at *p* < 0.05. All individual data points for each outcome of interest were visually inspected using scatter plots and boxplots to identify any obvious extreme outliers (i.e. exceeding 2.5 times the inter-quartile range). Between-scanner difference was calculated, and the same approach was used to identify extreme outliers. When implausible values were identified, each pair of scans were visually scrutinised to determine whether participant positioning was consistent, ROIs were in reasonable agreement, and that no appreciable motion artefacts were present on either scan. Descriptive statistics are presented as the mean and the standard deviation of the mean (SD), two-tailed paired t tests were applied to test for significant differences between measures obtained on the respective pairs of scanners. Overall bias was investigated visually with Bland Altman plots. Cross-calibration equations were produced using linear regression, where the independent variable was the outcome measure from the rural site, and the dependent variable was the same outcome measure from the urban site. The validity of the resulting equations was investigated by transforming bone and body composition data from the urban site and repeating paired t tests between the “corrected” urban scanners and the rural reference scanners. Bland Altman plots were also repeated using these adjusted data to determine if agreement improved following cross-calibration.

## Results

We recruited a total of 64 Gambian adults (58% M) aged 18–68 years old, for whom descriptive characteristics are presented in Table 1. Participants were scanned with a median [IQR] of 3[2;4] days between their scans at the urban and rural imaging facilities. Sixty-two participants had usable pairs of WB and TH scans available, while 61 pairs of scans were available at the lumbar spine. For iDXA body composition measures, complete data were available for 60 participants. One, 1 and 2 pairs of scans were excluded at the WB, TH and LS, respectively, due to different scan modes being used. pQCT scans at the distal (4%) and proximal (50%) tibia sites were available for 59 and 62 participants, respectively, after the exclusion of scans with movement artefact. Mean (SD) DXA and pQCT bone and body composition outcome measures for each scanning site are summarised in Table 2.

**Table 1:** Descriptive data of participants (*n* = 64) who participated in the study

Age (years)	30.9 (13.5)
Height (m)	1.69 (0.08)
Weight (kg)	62.2 (11.2)
BMI (kg/m^2^)	21.7 (4.0)
Time to follow up (days)	3 [2;4]

All data Mean (SD), except time to follow up Median [IQR]

**Table 2:** Bone and body composition values from DXA and pQCT at rural and urban Gambian research centres

Scan site	Outcome	Rural	Urban	*p* value
WB	aBMD (g/cm^2^) ^(*n*=62)^	**1.159 (0.126)**	**1.153 (0.124)**	** < 0.001**
	BMC (g) ^(*n*=62)^	2635 (458)	2633 (455)	0.369
	BA (cm^2^) ^(*n*=62)^	**2263 (214)**	**2272 (215)**	**0.002**
	LM (g) ^(*n*=61)^	**45938 (8087)**	**46320 (8133)**	** < 0.001**
	FM (g) ^(*n*=61)^	**13592 (8890)**	**13253 (8943)**	** < 0.001**
LS	aBMD (g/cm^2^) ^(*n*=61)^	1.150 (0.167)	1.148 (0.169)	0.556
	BMC (g) ^(*n*=61)^	65.14 (14.13)	65.12 (14.48)	0.963
	BA (cm^2^) ^(*n*=61)^	56.25 (6.36)	56.31 (6.41)	0.721
TH	aBMD (g/cm^2^) ^(*n*=62)^	1.133 (0.184)	1.133 (0.182)	0.851
	BMC (g) ^(*n*=62)^	37.71 (7.82)	37.69 (7.63)	0.823
	BA (cm^2^) ^(*n*=62)^	33.13 (2.95)	33.12 (2.91)	0.970
4% tibia	Tot.vBMD (mg/cm^3^) ^(*n*=59)^	**328.84 (58.60)**	**313.58 (54.82)**	** < 0.001**
	Tb.vBMD (mg/cm^3^) ^(*n*=59)^	**235.46 (49.30)**	**224.62 (45.76)**	** < 0.001**
	Tot.A 4% tibia (mm^2^) ^(*n*=59)^	1143.92 (172.60)	1148.28 (174.05)	0.436
50% tibia	Ct.vBMD (mg/cm^3^) ^(*n*=62)^	**1216.36 (31.15)**	**1144.88 (29.32)**	** < 0.001**
	BMC (mg/mm) ^(*n*=62)^	**361.51 (65.49)**	**336.77 (60.32)**	** < 0.001**
	Ct.A CSA (mm^2^) ^(n=62)^	**297.43 (54.73)**	**294.56 (54.60)**	** < 0.001**
	Ct.Th (mm) ^(*n*=62)^	4.65 (0.66)	4.68 (0.68)	0.075
	Tot.A 50% (mm^2^) ^(*n*=62)^	462.49 (79.39)	462.27 (78.14)	0.721
	SSI (mm^3^) ^(*n*=62)^	**2056.99 (484.57)**	**1969.27 (456.50)**	** < 0.001**
	CSMA (mm^2^) ^(*n*=62)^	3129.41 (684.35)	3110.50 (716.42)	0.470
	Fat CSA (mm^2^) ^(*n*=62)^	1191.29 (664.09)	1162.70 (692.19)	0.252
	Mu.Den (mg/cm^3^) ^(*n*=62)^	**73.03 (1.50)**	**71.71 (1.83)**	** < 0.001**

Values are mean (SD), bold indicates *p* < 0.05. *WB* whole body, *LS* lumbar spine, *TH* total hip, *aBMD* areal bone mineral density, *BMC* bone mineral content, *BA* mineral density, *Tb.vBMD* trabecular vBMD, *Tot.A* total area, *Ct.vBMD* cortical vBMD, *Ct.A* cortical area, *Ct.Th *cortical thickness, *CSA* cross-sectional area, *CSMA* cross-sectional muscle area, *SSI* stress–strain index, *Mu.Den* muscle density

### Between-Scanner Differences

WB aBMD and WB BA were significantly different between the two iDXA scanners; aBMD was lower on the urban scanner compared to the reference rural scanner; BA was higher on the urban scanner (*p* < 0.001 and *p* = 0.002, respectively) (Table 2.) and WB BMC did not differ between scanners. No significant between-scanner differences were found at the TH or LS for any measures of interest (Table 2). All DXA-derived body composition measures differed significantly between scanners, the urban scanner reported greater WB LM and lower FM compared to the reference scanner (both *p* < 0.001) (Table 2).

At the distal tibia, pQCT Tot.vBMD and Tb.vBMD measures were lower on the urban pQCT compared to the reference scanner (both *p* < 0.001), though bone geometry did not differ (Table 2). At the proximal tibia, Ct.vBMD, BMC, Ct.A and SSI were all lower on the urban scanner (all *p* < 0.001, Table 2). Of these the between-scanner difference in BMC was the greatest in magnitude with the urban scanner reporting approximately 7% higher than the rural, followed by Ct.vBMD where there was an approximately 6% difference in the same direction (Table 2). CSMA and Fat CSA did not differ between scanners but Mu.Den was lower on the urban pQCT scanner (*p* < 0.001, Table 2).

**Fig. 1:**
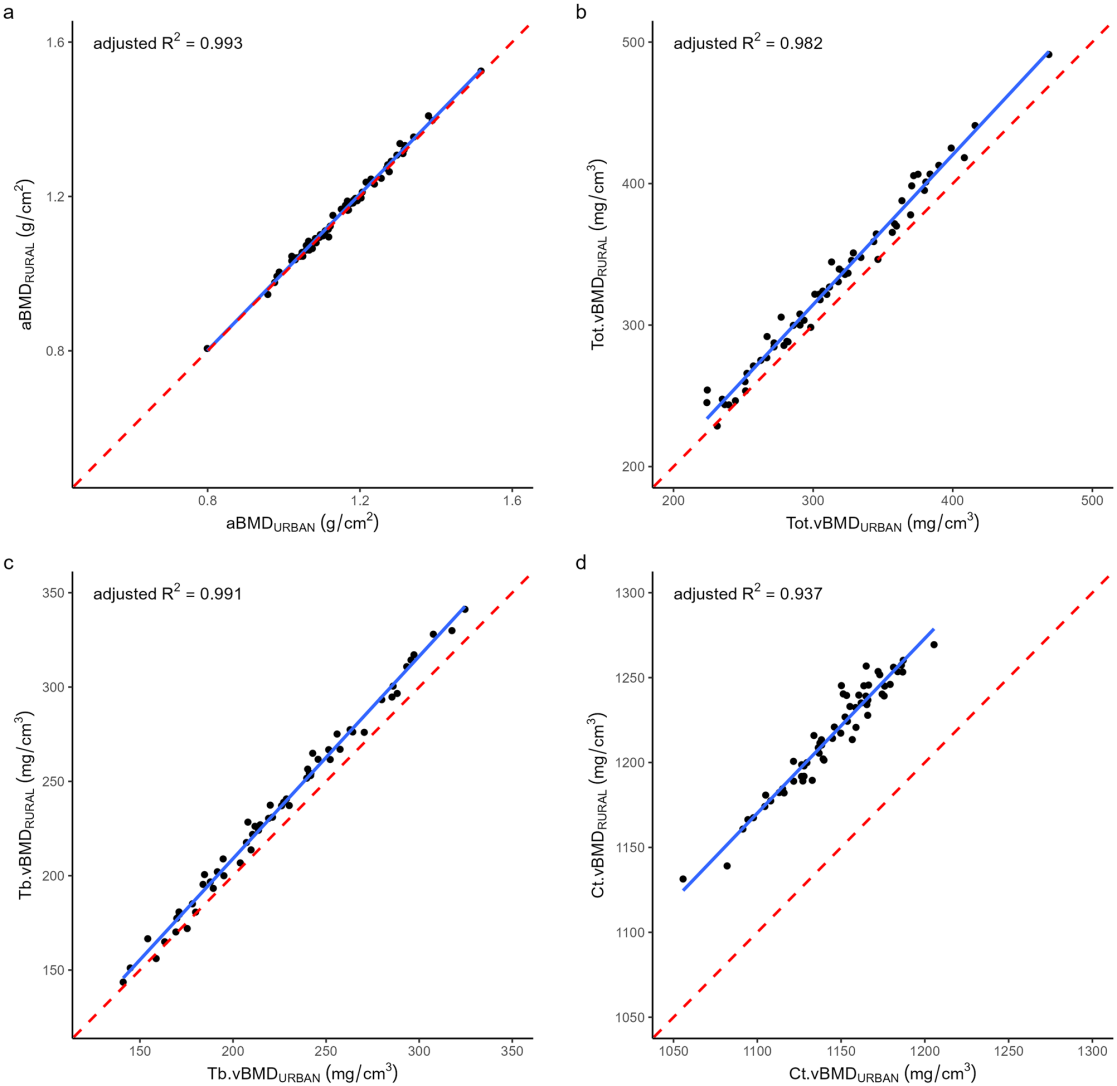
Correlation between rural and urban scanners for **a**) DXA WB aBMD, **b**) pQCT Tot.vBMD, **c**) pQCT Tb.vBMD, and **d**) pQCT Ct.vBMD. Adjusted R^2^ shown from linear model where the independent variable was the outcome measure from the rural site, and the dependent variable was the same outcome measure from the urban site. *DXA* Dual-energy X-ray absorptiometry; *pQCT* peripheral quantitative-computed tomography; *WB aBMD* whole body areal bone mineral density; *Tot.vBMD* total volumetric bone mineral density; *Tb.vBMD* trabecular volumetric bone mineral density; *Ct.vBMD* cortical volumetric bone mineral density

**Fig. 2:**
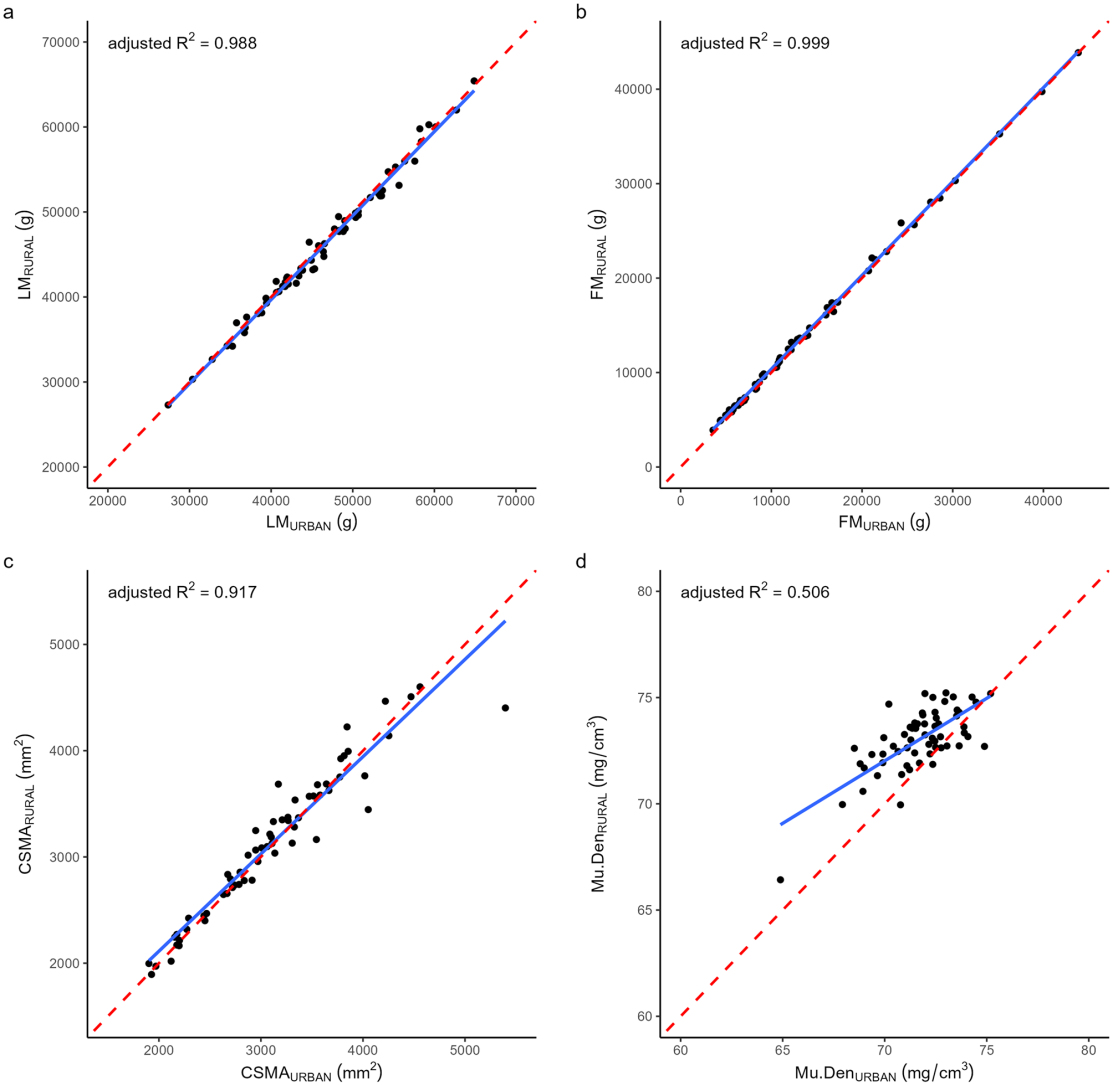
Correlation between rural and urban scanners for **a**) DXA WB LM, **b**) DXA WB FM, **c**) pQCT CSMA, **d**) pQCT Mu.Den. Adjusted R^2^ shown from linear model where the independent variable was the outcome measure from the rural site, and the dependent variable was the same outcome measure from the urban site. *DXA* Dual-energy X-ray absorptiometry; *pQCT* peripheral quantitative-computed tomography; *WB LM* whole body lean mass; *WB FM* whole body fat mass; *CSMA* cross-sectional muscle area; *Mu.Den* muscle density

**Fig. 3:**
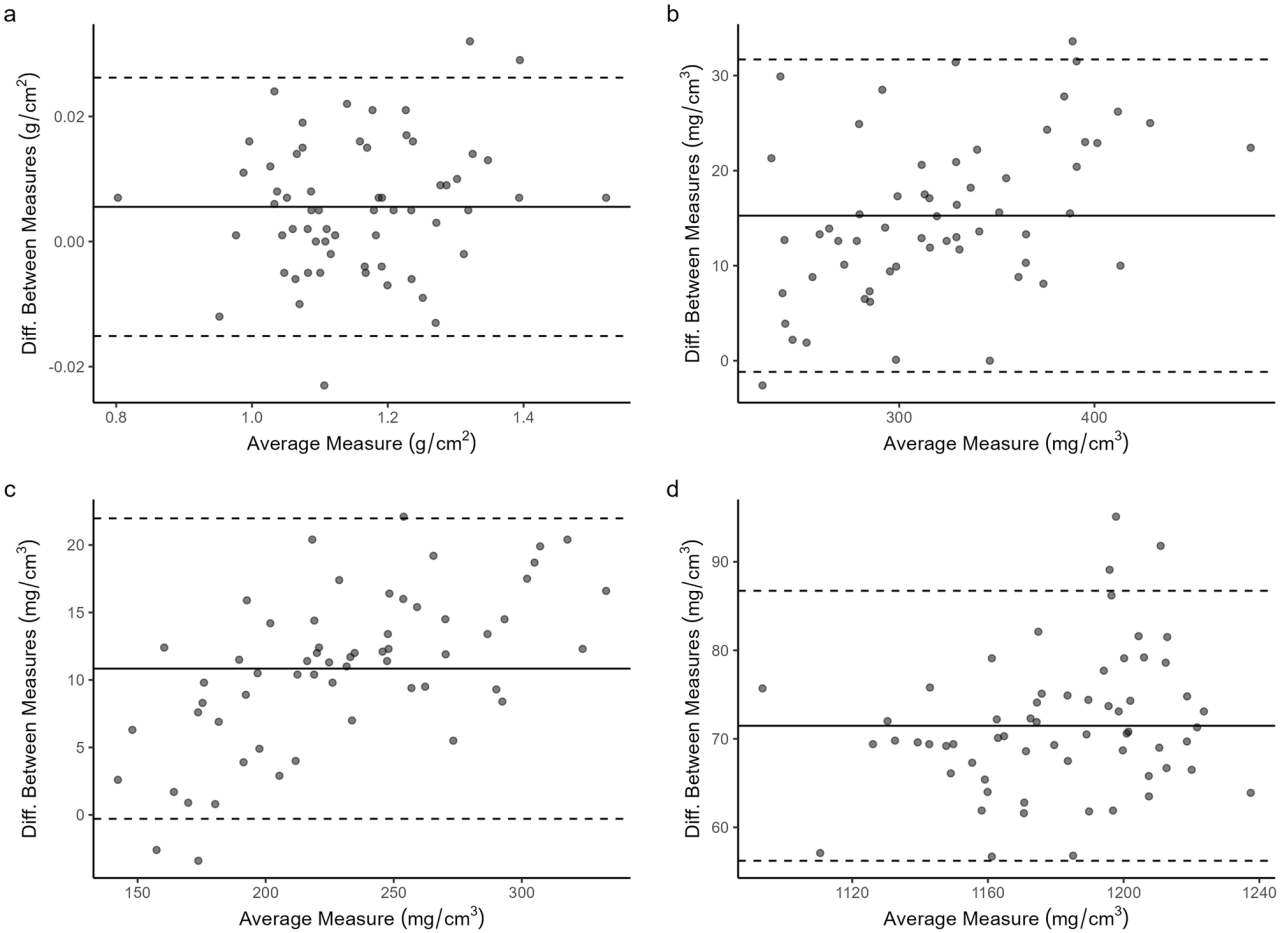
Bland–Altman plots for **a**) DXA WB aBMD, **b**) pQCT Tot.vBMD, **c**) pQCT Tb.vBMD, **d**) pQCT Ct.vBMD before the application of cross-calibration equations. DXA, Dual-energy X-ray absorptiometry; *pQCT* peripheral quantitative-computed tomography; *WB aBMD* whole body areal bone mineral density; *Tot.vBMD* total volumetric bone mineral density; *Tb.vBMD* trabecular volumetric bone mineral density; Ct.vBMD, cortical volumetric bone mineral density

**Fig. 4:**
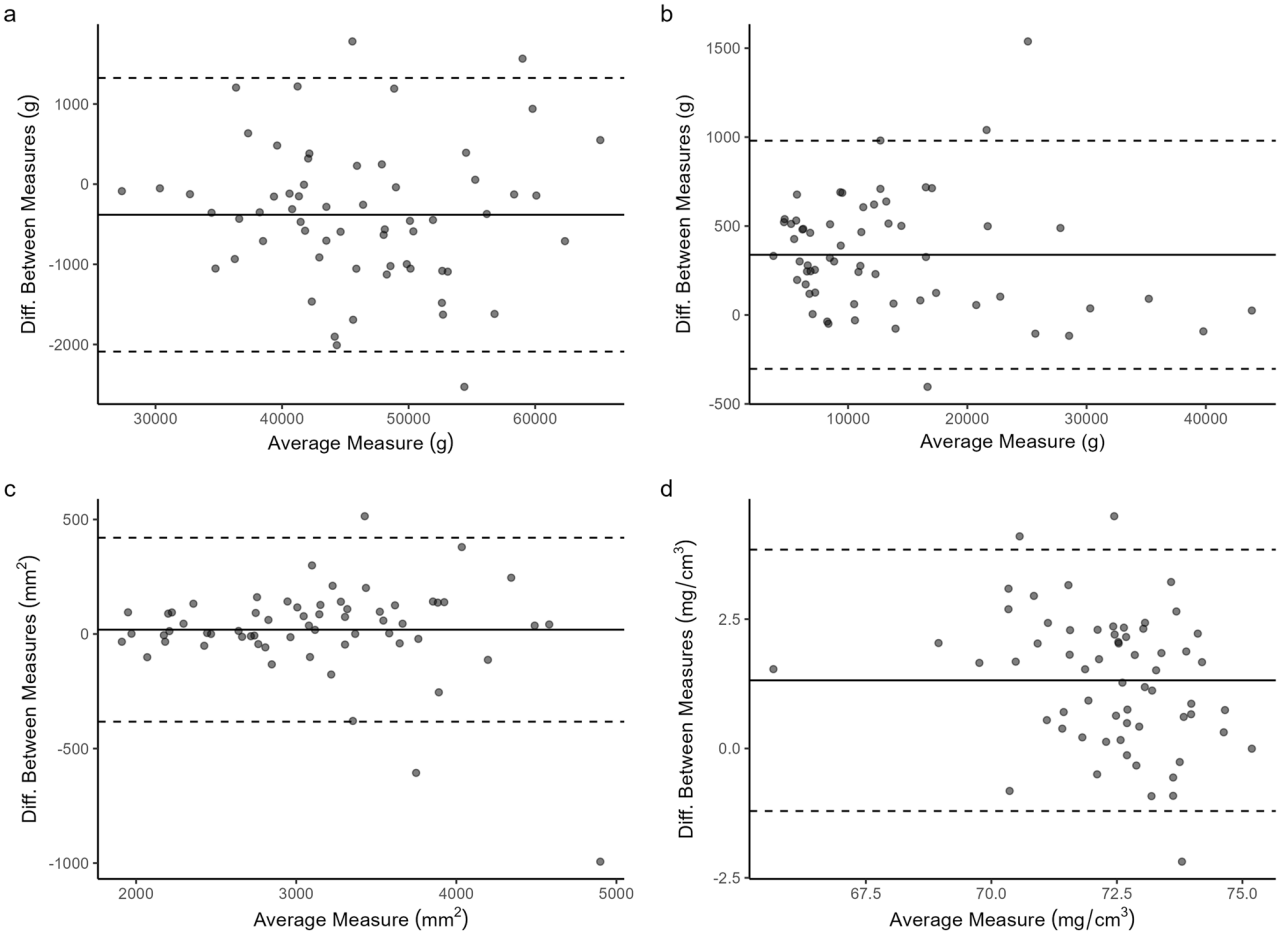
Bland–Altman plots for a) DXA WB LM, b) DXA WB FM, c) pQCT CSMA, d) pQCT Mu.Den. before the application of cross-calibration equations. Dual-energy X-ray absorptiometry; pQCT, peripheral quantitative-computed tomography; WB LM, whole body lean mass; WB FM, whole body fat mass; CSMA, cross-sectional muscle area; Mu.Den, muscle density

Figures 1 and 2 present correlation plots for select DXA and pQCT bone (Fig. 1) and body composition (Fig. 2) measures. In general, correlation was high between the respective pairs of scanners for all bone and body composition measures (Table 3), except for pQCT Mu.Den at the 50% tibia (*R*^2^ = 0.51, Fig. 2). Bland–Altman analysis indicated that greater bias was seen at higher densities, particularly for pQCT vBMD (Fig. 3). No such pattern of systematic bias was observed for body composition measures from either scanner pair (Fig. 4).

**Table 3:** Results from linear regression of urban against rural DXA and PQCT measurements to give cross-calibration equations for correction of urban to rural site data

Outcome		Intercept	Slope	R2	Cross-calibration equations
WB	aBMD	− 0.008(− 0.033, 0.017)	1.012 (0.990, 1.034)	99.3	1.012*WB aBMD_URBAN_− 0.008
	BMC	− 10.75 (− 45.01, 23.50)	1.005 (0.992, 1.018)	99.8	1.005*WB BMC_URBAN_− 10.75
	BA	15.15 (-42.68, 72.98)	0.990 (0.964, 1.015)	99.0	0.990*WB BA_URBAn_ + 15.15
	LM	147.50 (− 1156.59, 1451.59)	0.989 (0.961, 1.016)	99.8	0.989*LM_URBAN_ + 147.50
	FM	426.07 (276.56, 575.58)	0.993 (0.984, 1.003)	99.9	0.993*FM_URBAN_ + 426.07
LS	aBMD	0.032 (− 0.016, 0.081)	0.974 (0.932, 1.016)	97.3	0.974*LS aBMD_URBAN_ + 0.032
	BMC	2.50 (− 0.34, 5.34)	0.962 (0.919, 1.004)	97.2	0.962*LS BMC_URBAN_ + 2.50
	BA	1.39 (− 1.46, 4.24)	0.974 (0.924, 1.025)	96.2	0.974*LS BA_URBAN_ + 1.39
TH	aBMD	− 0.011 (− 0.030, 0.009)	1.009 (0.992, 1.026)	99.6	1.009*TH aBMD_URBAN_− 0.011
	BMC	− 0.75 (− 1.72, 0.21)	1.021(0.995, 1.046)	99.1	1.021*TH BMC_URBAN_− 0.75
	BA	0.44 (− 1.56, 2.44)	0.987 (0.927, 1.047)	94.6	0.987*TH BA_URBAN_+0.44
4% tibia	Tot.vBMD	-3.42 (-15.31, 8.47)	1.060 (1.022, 1.097)	98.2	1.060*total vBMD_URBAN_ − 3.42
	Tb.vBMD	− 5.50 (− 11.61, 0.61)	1.072 (1.046, 1.099)	99.1	1.072*trabecular vBMD_URBAN_ − 5.50
	Tot.A	39.70 (− 34.94, 114.34)	0.962 (0.897, 1.026)	93.9	0.962* Total CSA_URBAN_+39.70
50% tibia	Ct.vBMD	37.99 (− 40.03, 116.00)	1.029 (0.961, 1.097)	93.7	1.029* Ct.vBMD_URBAN_+37.99
	BMC	− 3.38 (− 9.65, 2.88)	1.083 (1.065, 1.102)	99.6	1.083* BMC_URBAN_ − 3.38
	Ct.A	2.87 (− 2.63, 8.37)	1.000 (0.982, 1.018)	99.5	1.000* Ct.A_URBAN_+2.87
	Ct.Th	0.16 (− 0.05, 0.38)	0.959 (0.914, 1.004)	96.8	0.959* Ct.Th_URBAN_+0.16
	Tot.A	− 6.37 (− 13.42, 0.67)	1.014 (0.999, 1.029)	99.7	1.014* Total CSA_URBAN_ − 6.37
	SSI	9.83 (− 102.25, 121.90)	1.040 (0.984, 1.095)	95.8	1.040*SSI_URBAN_+9.83
	CSMA	282.20 [57.16, 507.23]	0.915 [0.845, 0.986]	91.7	0.915*CSMA_URBAN_+282.20
	Fat CSA	120.89 [26.74, 215.03]	0.921 [0.851, 0.990]	92.0	0.921* Fat CSA_URBAN_+120.89
	Mu.Den	30.83 [20.24, 41.42]	0.588 [0.441, 0.736]	50.6	0.588*Mu.Den_URBAN_+30.83

Urban values treated as the independent variable, and rural values as the dependent variable in each model. Values are mean (95% CI), bold indicates *p* < 0.05. *WB* whole body, *LS* lumbar spine, *TH* total hip, *aBMD* areal bone mineral density, *BMC* bone mineral content, *BA* bone area, *LM* lean mass, *FM* fat mass, *Tot.vBMD* total volumetric bone mineral density, *Tb.vBMD* trabecular vBMD, *Tot.A* total area, *Ct.vBMD* cortical vBMD, *Ct.A* cortical area, *Ct.Th* cortical thickness, *CSA* cross-sectional area, *CSMA* cross-sectional muscle area; *SSI* stress strain index, *Mu.Den* muscle density

### Cross-Calibration

Table 3 details the results of linear regression analyses used to generate cross-calibration equations for all measures of interest. Applying these equations to those values which differed significantly at the outset (Table 2) attenuated all statistically significant differences between the respective scanner pairs (Table S1). This was further supported by the repeated Bland–Altman plots where no clear systematic bias was evident after the transformation of the urban scanner data (Figure S1, Figure S2).

## Discussion

This paper presents our experience of cross-calibrating DXA and pQCT scanners at two bone imaging facilities over 150 km apart in sub-Saharan Africa. Several between-scanner differences were identified in both the pQCT and DXA pairs. This was most apparent for density-based outcomes, with greater differences emerging at higher densities particularly in the case of pQCT (i.e. cortical vBMD). With the exception of BMC, between-scanner differences were apparent for all DXA-measured whole body bone and body composition outcomes though their magnitude was lower compared to pQCT. Cross-calibration equations from the in vivo participant data attenuated all differences between scanners. Importantly, participants were representative of the populations usually imaged at these two research facilities meaning that the corrections should be applicable in our context. While studies have been published detailing the cross-calibration of different modalities in hospital or research settings, to date these have been from high income settings [4, 8, 13, 14, 19–26]. To the best of our knowledge, this represents the first such study in sub-Saharan Africa, where research sites with dedicated bone imaging facilities are few, and hospitals have limited access to standard clinical modalities such as DXA.

This work is of particular importance to our ongoing research in The Gambia. Due to the COVID-19 pandemic, travel restrictions, and the related disruption of global supply chains, the availability of replacement parts in the event of scanner failure has become more difficult. In the event of scanner failure or downtime, scanning in ongoing research studies may be delayed due to the logistics of procuring replacement parts. When cross-calibration with neighbouring research centres has been performed, it may be possible to transport participants between sites, obtain scheduled scans, and subsequently adjust the data using cross-calibration equations. This work also supports our research into the ongoing demographic transition and urbanisation taking place in The Gambia, which has resulted in many of our participants in longitudinal studies migrating from rural to urban area. An additional benefit of this cross-calibration is that we can control for potential between-scanner differences when making comparisons of geographically distinct populations (e.g. urban vs rural dwellers) who may have distinct musculoskeletal phenotypes. Our experiences and findings as such may be generalisable to other resource-limited settings where the logistics of sourcing parts and in-country repair may result in lengthy scanner downtime. In our context, this issue is mitigated by our ability to obtain scans at the other research facility and cross-calibrate the data as appropriate.

Perhaps unsurprisingly we found no significant between-scanner differences in DXA measures at the total hip and lumbar spine skeletal sites routinely used for clinical reporting and the diagnosis of osteoporosis. We pragmatically chose to analyse TH rather than a femoral neck region of interest as the relatively short hip axis length typical of this population [16] often makes it difficult to place a region of interest accurately without inadvertently including other bone tissues. This difficulty is further increased when trying to do so in longitudinal studies. Between-scanner differences from the whole body scans may reflect the difficulties in calibrating scanners for a range of densities though could conceivably be the result of subtle differences in positioning, or hydration status for body composition. While we cannot discount entirely the potential effects of positioning, participant hydration or clothing on these outcome measures all scans at both facilities were obtained in line with the same strict standard operating procedures and the same operating staff at both sites.

Although the acquisition of longitudinal pQCT scans over the region of interest is more subject to differences in limb rotation and positioning than DXA, the magnitude of between-scanner differences we encountered far exceeded the CV values for the individual instruments. Between-scanner differences were greatest in number and magnitude for density or density-derived measures at both trabecular- and cortical-rich sites. Mu.Den was also similarly affected. While we have successfully used the European Forearm Phantom (EFP)[13] in previous studies to correct for pQCT vBMD [12], the design of this specific phantom does not allow for cross-calibration of body composition and as such has not previously allowed us to correct Mu.Den. All Bland–Altman plots of pQCT vBMD consistently illustrated that the mean difference between scanners were most pronounced at higher densities (Fig. 3, Figure S1). For body composition measures, such bias was not readily apparent from the Bland–Altman plots despite the noted between-scanners differences and low correlation for Mu.Den.

### Strengths and Limitations

These are the first multi-site cross-calibration data for pQCT and DXA to be published in sub-Saharan Africa. In vivo cross-calibration allowed for a greater range of bone densities, applicable to our study population, to be included rather than a phantom. This approach also allowed cross-calibration of muscle and fat measures since there are currently no phantoms widely used for pQCT body composition outcomes. While a variety of established and novel DXA phantoms [7, 8, 27, 28] are available capable of representing different body compartments, it has been shown that often used encapsulated spine phantoms do not adequately cross-calibrate densitometers for body composition measurement [7]. Furthermore, the practicalities of procuring and transporting such phantoms present a barrier to their widespread use in SSA. Additionally, we endeavoured to ensure that participants reflected the population we recruit in our studies, making the cross-calibrations appropriate for our research context. This allows us to avoid applying cross-calibration equations obtained in other ethnic groups primarily from HIC in North America and Europe, which may not be comparable to The Gambia. We had high retention in the study and very few scans were excluded from analysis due to common issues such as movement artefacts. A limitation to our study is that participants were not scanned on the same day on both scanner pairs. Given the large distance between the two sites, we pragmatically aimed to scan participants at both facilities within 3 days of the incident scan. Biological changes in the skeleton during such a narrow window are unlikely and the ability to detect any changes would be so small in magnitude that the scanners would be unlikely to detect them. We acknowledge that hydration status could impact measures of body composition at either, or both, of the scan visits though despite use of standardised protocols.

## Conclusions

Between-scanner iDXA differences were not present at routinely scanned clinical sites (i.e. hip and lumbar spine) but were identified for whole body measures of aBMD, lean mass and fat mass. Following the derivation and application of cross-calibration equations, these differences were fully attenuated. A greater number of pQCT bone measures, particularly measures of vBMD or their derivatives (i.e. bone strength), were found to differ significantly prior to correction, with measures from the cortical compartment being most affected. Again, in vivo cross-calibration corrected these differences. pQCT measured muscle density was also successfully corrected by cross-calibration. This highlights the importance of cross-calibration of devices between facilities where possible with participants or phantoms where not feasible. Our experiences and findings as such may be generalisable to other resource-limited settings where the logistics of sourcing parts and in-country repair may result in lengthy scanner downtime.

## Supplementary Information

Below is the link to the electronic supplementary material.

Supplementary file1 (DOCX 388 kb)
