# Supplementary material for: Cross-Calibration of iDXA and pQCT Scanners at Rural and Urban Research Sites in The Gambia, West Africa
Source: Calcif Tissue Int. 2023 Mar 2;112(5):573–83. doi: 10.1007/s00223-023-01071-6 (PMC9977475; doi:10.1007/s00223-023-01071-6)

| Table S1: Bone and body composition values from DXA and pQCT at rural and urban Gambian research centres after application of cross-calibration-equations in Table 3. Outcomes where there was no statistically significant difference in Table 2 were not transformed. | | | | | | |
| --- | --- | --- | --- | --- | --- | --- |
| **Site** | **Outcome** | | **Rural** | **Urban-transformed** | **p-value** |  |
| WB | | aBMD (g/cm^2^) ^(n=62)^ | 1.159 (0.126) | 1.159 (0.126) | 0.825 |  |
|  |  | BMC (g) ^(n=62)^ | - | - | - |  |
|  |  | BA (cm^2^) ^(n=62)^ | 2263 (214) | 2265 (213) | 0.530 |  |
|  | | LM (g) ^(n=61)^ | 45938 (8087) | 45958 (8043) | 0.855 |  |
|  | | FM (g) ^(n=61)^ | 13592 (8890) | 13587 (8880) | 0.909 |  |
| 4% tibia | | Tot.vBMD (mg/cm^3^) ^(n=59)^ | 328.84 (58.60) | 328.98 (58.11) | 0.893 |  |
|  |  | Tb.vBMD (mg/cm^3^) ^(n=59)^ | 235.46 (49.30) | 235.30 (49.05) | 0.778 |  |
|  |  | Tot.A (mm^2^) ^(n=59)^ | - | - | - |  |
| 50% tibia | | Ct.vBMD (mg/cm^3^) ^(n=62)^ | 1216.36 (31.15) | 1216.07 (30.17) | 0.770 |  |
|  |  | BMC (mg/mm) ^(n=62)^ | 361.51 (65.49) | 361.35 (65.32) | 0**.**767 |  |
|  |  | Ct.A (mm^2^) ^(n=62)^ | 297.43 (54.73) | 297.43 (54.60) | 0.998 |  |
|  |  | Ct.Th (mm) ^(n=62)^ | - | - | - |  |
|  |  | Tot.A (mm^2^) ^(n=62)^ | - | - | - |  |
|  |  | SSI (mm^3^) ^(n=62)^ | 2056.99 (484.57) | 2057.88 (474.76) | 0.943 |  |
|  | | CSMA (mm^2^) ^(n=62)^ | - | - | - |  |
|  | | Fat CSA (mm^2^) ^(n=62)^ | - | - | - |  |
|  | | Mu.Den (mg/cm^3^) ^(n=62)^ | 73.03 (1.50) | 73.00 (1.08) | 0.803 |  |
| Values are mean (SD), bold indicates p<0.05. . WB, whole body; LS, lumbar spine; TH, total hip; aBMD, areal bone mineral density; BMC, bone mineral content; BA, bone area; LM, lean mass; FM, fat mass; Tot.vBMD, total volumetric bone mineral density; Tb.vBMD, trabecular vBMD; Tot.A, total area; Ct.vBMD, cortical vBMD; Ct.A, cortical area, Ct.Th, cortical thickness; CSA, cross-sectional area; CSMA, cross-sectional muscle area; SSI, stress strain index; Mu.Den, muscle density. | | | | | | |

Figure S1


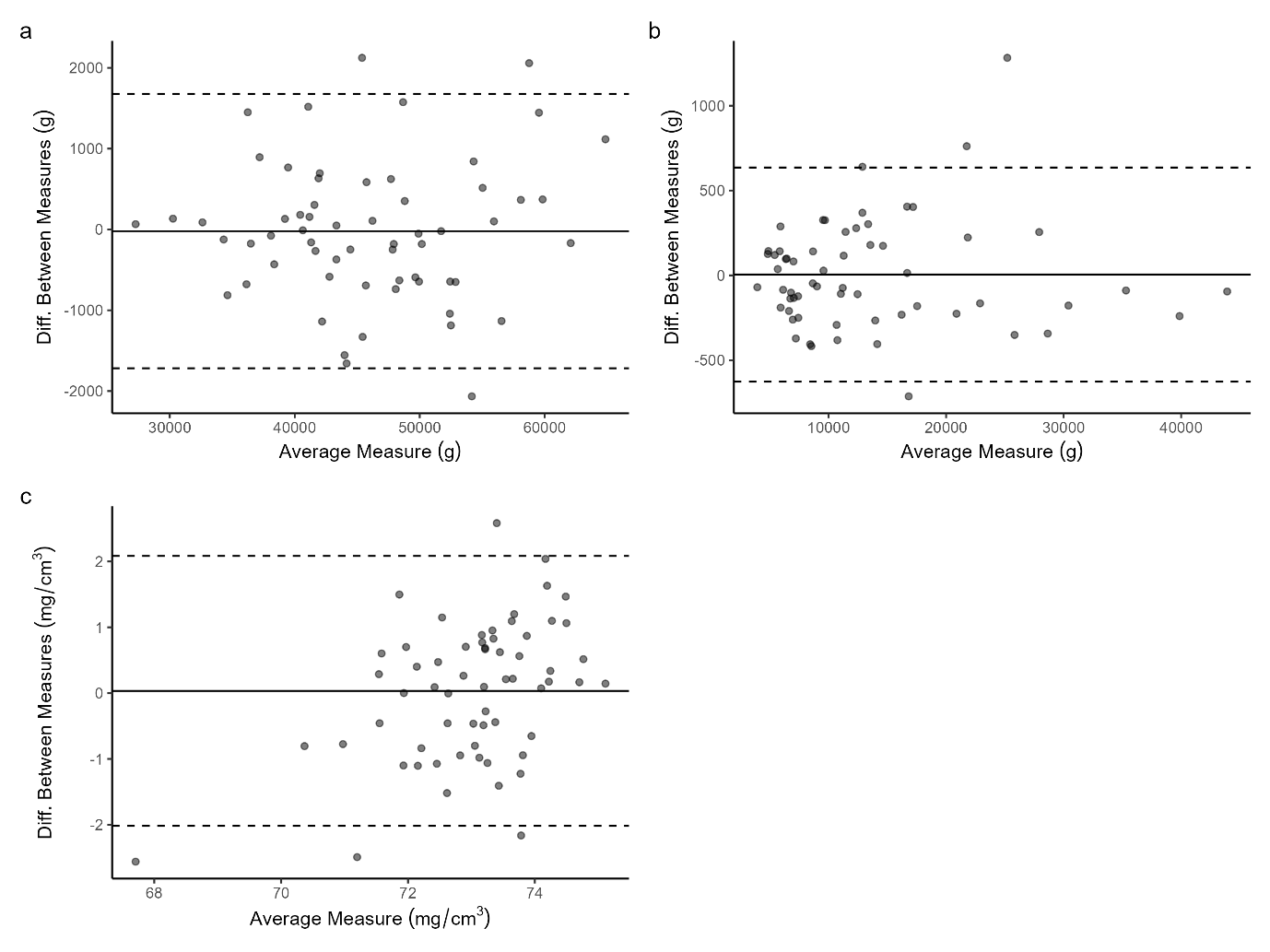


Figure S2
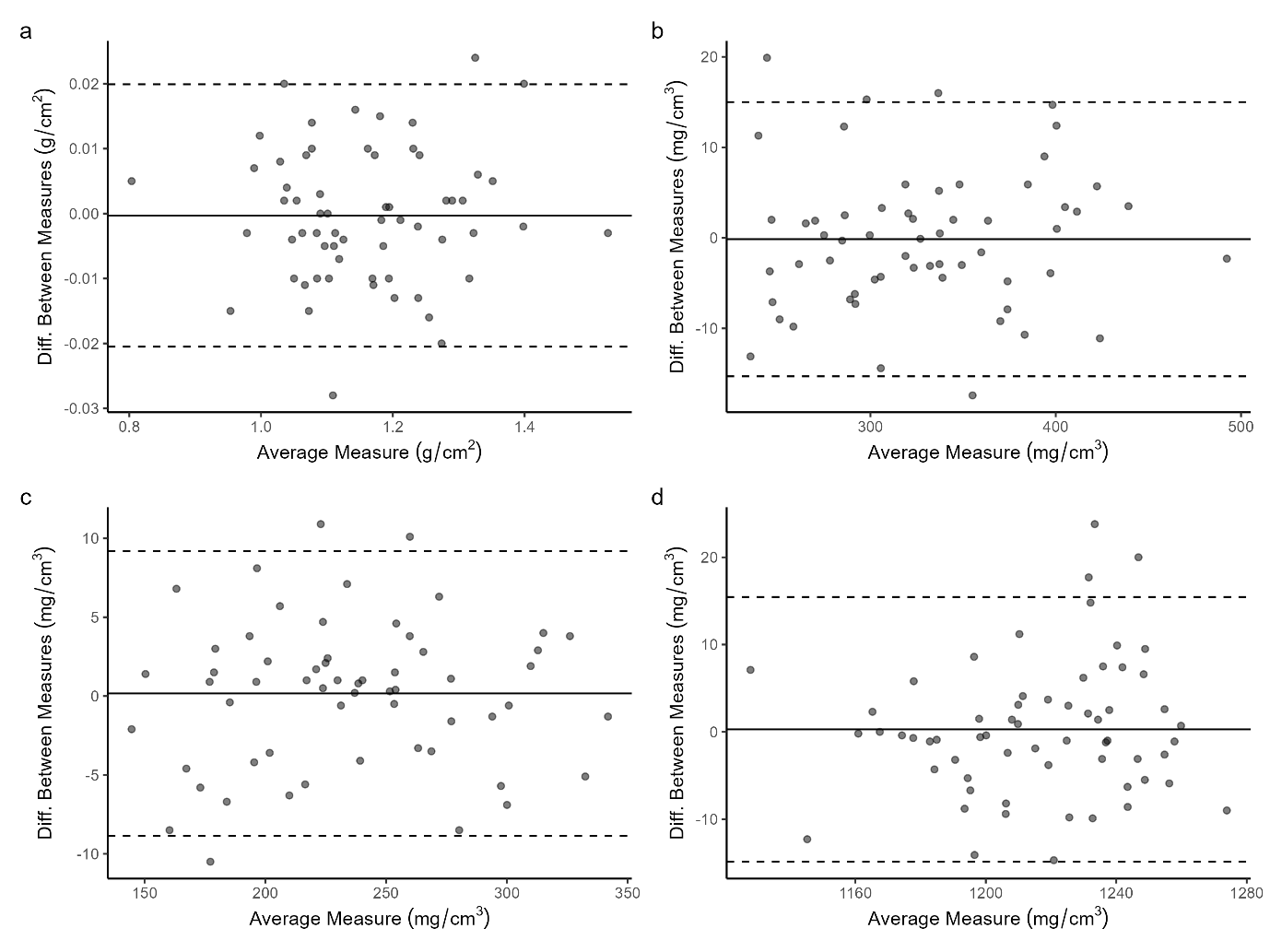

Supplement: Supplementary file 1 — Supplementary file1 (DOCX 388 kb) [file 223_2023_1071_MOESM1_ESM.docx]
